# Emotional Eating and Obesity: An Update and New Insights

**DOI:** 10.1007/s13679-025-00661-9

**Published:** 2025-09-29

**Authors:** Abby Braden, Erica Ahlich, Afton M. Koball

**Affiliations:** 1 Department of Psychology, Bowling Green State University, 822 East Merry Avenue, Bowling Green, OH USA; 2 Department of Psychology, University of South Alabama, Mobile, AL USA; 3 Department of Psychiatry and Psychology, Mayo Clinic, Rochester, MN USA

**Keywords:** Obesity, Emotional eating, Emotion regulation, Behavioral weight loss

## Abstract

**Purpose of Review:**

The current review aimed to (1) synthesize information regarding the association between emotional eating (EE) and BMI, as well as between EE and dietary intake and psychological symptoms; (2) describe factors thought to underlie and/or maintain EE; and (3) summarize recent evidence supporting behavioral treatments of EE in people with obesity.

**Recent Findings:**

Adults with obesity frequently report EE. Emotion regulation and learning principles are key variables that may influence EE in adults with obesity. Behavioral treatments show promise for decreasing EE, in the short-term, especially cognitive-behavioral and mindfulness-based approaches.

**Summary:**

Although psychosocial factors are critical to the understanding of EE mechanisms and treatment, limitations include measurement of EE and construct definitions of proposed theoretical variables. Additionally, behavioral interventions overlap which obscures the relative utility of treatment components. Future work should clarify causal mechanisms of EE in the context of obesity to inform treatment development.

## Introduction

Contemporary science emphasizes the value of examining obesity subtypes given heterogenous etiological contributors to the disease [1]. Emotional eating (EE) is one obesity subtype that has generated a great deal of interest from researchers and clinicians [2, 3]. When asked, roughly 50–57% [4, 5] of adults with obesity endorse EE and early data suggests a strong link between EE and BMI [6]. Therefore, EE is likely a relevant behavioral intervention target for a notable proportion of adults with obesity. The present review focuses on the overlap between EE and obesity, with an emphasis on dietary and psychological correlates, psychosocial etiological/maintenance factors, and the impact of behavioral treatments on EE.

EE is most often defined as eating that is triggered by negative emotions as opposed to genuine physiological hunger [7, 8]. EE is associated with disordered eating, depression, and other maladaptive psychological experiences [9]. EE can be present in clinically diagnosed eating disorders, but EE is not a diagnostic symptom [10]. EE is positively related to binge eating and binge eating disorder (BED) [11]; however, these terms have distinct definitions. BED is characterized by eating an objectively large quantity of food, while experiencing a loss of control, in the context of additional psychological symptoms [10]. Although negative emotions frequently precede binge eating, binge episodes can also be triggered by other factors as emotional triggers are not required for a BED diagnosis. Moreover, emotional eating is not defined by the amount of food consumed, nor eating speed. One study of college-aged women attempted to distinguish between EE, overeating, and binge eating [12]. A large association was observed between EE and binge eating; however, in the follow-up analysis, EE only predicted the number of overeating and binge eating episodes among women with at least one episode of overeating or binge eating over the course of the 3-day monitoring period. The authors speculated that EE may be a marker of more severe disordered eating. EE and binge eating also share theoretical underpinnings, and some researchers have considered the possibility that they exist on a “continuum” [13].

In a seminal review from 1989, Ganley [14] asserted that EE is frequently experienced by people with obesity and occurs across social class but is more common in women than men. In addition, Ganley [14] indicated that a variety of emotions prompt eating and that these differ across people. Boredom, stress, depression, anxiety, tiredness, and anger have been identified as emotions that frequently prompt eating [15]. One of the most often used self-report measures of EE, the Emotional Eating Scale (EES [8]), allows for the measurement of specific, negative emotions (i.e., anxiety, depression, and anger/frustration) which includes boredom, in its updated version [16]. Research has focused on determining negative emotions that are most likely to elicit EE behavior. In fact, boredom may be the most common trigger, as identified in college students [16] and adult men seeking bariatric surgery [15]. In another more recent clinical sample of adults with overweight/obesity, boredom, anxiety, and loneliness were the most frequent emotions reported to trigger eating, although loneliness was more common among those participants who had BED compared to those who did not [17]. Depression has also been identified as the most common emotion to prompt eating, endorsed by 44.4% of a treatment-seeking sample of adults who identified as emotional eaters [18].

Theoretical research on EE as a proposed contributor to obesity can be traced back to the mid-20th century. Much of this theoretical work proposes that emotions trigger overeating. Early theorists adopted a psychosomatic model, claiming that eating serves to regulate affect. In fact, Kaplan and Kaplan [19] argued that most cases of obesity are the result of anxiety that promotes overeating. Likewise, Bruch [20] argued that overeating occurs due to an inability to distinguish feelings of hunger from emotional distress. Other central theories of eating behavior, including restraint [21] and escape theories [22] emphasize the role of affect (see Canetti and colleagues [23] for a review of the link between emotions and eating). Building upon these prior theories, Macht [24] broadened the conceptual understanding of how food and emotions are related and argued that distinct emotions cause people to eat more or less, and that people vary in how they respond. Macht [24] also proposed that affect regulation theory is consistent with traditional behavioral paradigms in that emotions act as stimuli prompting eating, and eating is reinforced via operant conditioning. (i.e., decreased negative emotions).

Theoretical research has also emphasized the type of foods that usually coincide with EE, labeling them as “comfort foods” [25]. Reviews by Gibson [25, 26] summarize human and animal studies showing that stress leads to a preference for sweet, high-fat foods via suppression of the hypothalamic pituitary adrenal axis, mitigating stress and negative emotion. This can lead to overreliance on energy-dense foods, overeating, future weight gain, and eventually obesity. Taken together, early theoretical research has historically highlighted EE as a factor that likely contributes to obesity via overeating. Interestingly, in the decades that have followed, empirical investigations of EE, most of which rely on self-report measures, typically have not assessed the amount or type of food consumed when measuring EE. Instead, actual measurement of EE often examines emotions as they prompt the “desire” [7] or “urge” [8] to eat. It should also be noted that obesity is a complex condition, with substantial evidence that biological underpinnings interact with psychological and social factors to confer risk [27]. Some individuals with obesity do not experience elevated EE, and EE can be present in individuals of varying body weights [28].

The overarching goal of the current review is to provide a synthesis of recent literature on EE and obesity. For the purposes of this review, the focus will be solely on eating in response to negative, as opposed to positive emotions, given prior research suggesting that the latter is not as strongly associated with well-being, disordered eating, or emotion dysregulation [9]. The present review will also focus primarily on literature specific to EE, rather than binge eating. When included studies examined EE in the context of binge eating or findings specific to binge eating that may be pertinent to EE, it will be noted. The impact of EE on weight loss outcomes is also deemed beyond the scope of the present review and has been previously reviewed [29]. An updated review will provide researchers and clinicians with more current, evidence-based guidance about the significance and public health impact of EE in the context of obesity. New insights related to EE in obesity will also be highlighted. Aim 1 was to examine whether contemporary findings align with previous literature showing that individuals with obesity are at elevated risk for EE [14] and to describe associations between EE, dietary intake, and psychological symptoms in people with obesity. Dietary intake and psychological symptoms were examined to remain consistent with the behavioral focus of the review and summarize novel, recently published results that address these constructs [30, 31]. Aim 2 was to synthesize the latest findings regarding psychosocial variables that may underlie and/or maintain EE among people with elevated BMI. Lastly, Aim 3 was to summarize modern research on the impact of behavioral treatments on EE in people with obesity.

### Aim 1: Association between Obesity and EE

Recent research supports the long-held conviction that EE and obesity are strongly, positively related. In their 2018 review, Frayn & Knauper [29] provided strong preliminary evidence of this link between EE and body weight, based on longitudinal and intervention studies, although they acknowledge that effect sizes vary. Results of newly published systematic reviews add to this accumulation of evidence [30, 32]. In their review, Dakanalis and colleagues [30] restricted inclusion to articles published in the last decade, in an effort to summarize contemporary findings. Results, drawn from cross-sectional and prospective studies, showed a consistent pattern in which elevated EE was associated with higher BMI. Another recent meta-analysis concluded that adults with obesity reported higher levels of EE than adults with healthy weight status, but no differences in EE were observed between overweight and healthy weight categories [32].

Most research examining the association between EE and BMI is based on cross-sectional findings. In a cross-sectional study of 600 Italian young adults, EE and BMI were significantly associated but the effect was small [33]. Longitudinal studies also exist. Six prospective cohort studies conducted over the last 15 years, spanning Asia [34], North America [35], and Europe [36–39], showed that baseline EE was predictive of greater weight gain over time [40], although small effect sizes are generally observed. In one study of 3735 Finnish adults, EE was positively associated with laboratory-measured BMI and waist circumference 7 years later [39]. The same pattern was observed in a sample of 39,771 adults from an ongoing prospective study in France in which EE was positively associated with self-reported BMI, over an average of a 5-year follow-up [38]. Both studies found that the relationship between EE and BMI was stronger in women than men [38, 39]. In contrast to the above findings, one study from 2006 [41] did not find a significant longitudinal relationship between EE and BMI.

Established cut-offs on EE measures do not exist, making it difficult to quantify the prevalence of EE. Nonetheless, a recently published meta-analysis examined EE in the context of obesity or weight gain by analyzing studies that reported the frequency of EE, as measured with a validated psychometric questionnaire [42]. Results revealed a prevalence rate of 44.9% (95% CI: 29–62%) but heterogeneity was observed, based on the type of EE measure used. In a sample of 1626 Turkish adults, elevated EE was most common among those with obesity (43.5%) compared to individuals classified as overweight (37.2%), healthy weight (33.5%), and underweight (18.4%; of note, height and weight were self-reported, and data were collected during the global pandemic) [43]. EE rates likely vary based on the definition of EE and may be higher in clinical compared to convenience samples. In a sample of 387 Australian patients seeking outpatient obesity treatment, 58% of the sample were categorized as emotional eaters given that they endorsed at least a “small desire to eat” in response to negative emotions [44]. In another, smaller sample of U.S. adults (*n* = 63) seeking EE and weight loss treatment, a more stringent cut-off for elevated EE was used (i.e., “strong desire to eat”), and 44.4%, 20.6%, and 17.5% endorsed high levels of EE in response to depression, anxiety/anger, and boredom, respectively [18]. Given that these participants were seeking treatment for EE, it is not surprising that notable proportions of the sample were classified as emotional eaters.

#### Dietary Intake and Common Psychological Difficulties

The Dakanalis and colleagues review [30] and a recently published review by Gonzalez and colleagues [31] summarize studies that examined associations between dietary practices and EE. Both reviews concluded that EE is associated with consumption of energy-dense foods in males and females, across the lifespan [30, 31]. Examples of this association are present in recently published studies. In a sample of 303 women with overweight/obesity from Iran, EE was positively associated with energy intake and consumption of certain high-calorie foods such as muffins, cakes, pastries, ice cream, and candy [45]. Poor dietary choices are one likely behavioral explanation for the link between obesity and EE. For example, consumption of energy-dense foods partially mediated the association between EE and overeating in a Latino sample from the U.S., 78% of whom had obesity [46]. Betancourt-Núñez and colleagues [47] examined dietary patterns of emotional eaters among people with and without abdominal obesity. Results showed that having high levels of EE and abdominal obesity was associated with greater intake of snacks and fast food, sodium, and saturated fats and lower intake of fiber, folic acid, vitamins B and C. Interestingly, this same pattern was not observed in individuals who had abdominal obesity but not EE or in those with EE without abdominal obesity. Consequently, adults with obesity who engage in EE may be the most prone to unhealthy dietary behaviors. However, it is also important to recognize the potential role of social determinants of health, such as socioeconomic status and food availability, in emotional eating, diet, and obesity.

In addition to unhealthy dietary intake, adults with obesity and EE are likely to experience a sequela of co-existing psychological concerns. For example, in Dakanalis and colleagues’ review [30], a pattern of positive associations between EE and depression and anxiety/stress emerged. Much of the research examining depression and EE has considered EE as a mechanism, leading to unhealthy dietary behaviors and weight gain. In a cross-sectional analysis of Dutch adults, EE was a mediator, linking depression and consumption of fast-food/savory snacks [48]. Furthermore, studies have shown that the relationship between depression and BMI measured cross-sectionally [49], 5 [37] and 7 [39] years later, is partially explained by EE. In the cross-sectional study, 23.1% and 25.0% of the total effect was due to the indirect effect of depression via EE on BMI, in men and women, respectively [49]. It should be noted that these studies did not account for other possible mediating factors such as use of psychotropic medications. Psychological difficulties associated with EE seem to be independent of the effects of obesity. For example, in a sample of young adults from Italy, subgroup analyses showed that depression/anxiety symptoms were related to greater EE among individuals with healthy weight and overweight [33]. In other studies, EE was positively related to depression diagnosis and severity [48] and depression, anxiety, and stress, after controlling for BMI [50]. In addition to links between EE and depression/anxiety, adults with EE and obesity are likely at risk for clinically significant disordered eating. A recent meta-analysis revealed that the relationship between EE and disordered eating behaviors (e.g., dietary restraint, food addiction, binge eating, purging/vomiting) was stronger among people with a higher BMI [51].

### Aim 2: Mechanistic and Maintaining Factors

As discussed in the introduction, several frameworks have been offered through which to understand the mechanistic and moderating factors involved in EE among people with obesity. Rather than discussing each model in depth, Table 1 includes definitions of transtheoretical psychosocial factors, proposed links with EE, and citations of relevant, recent empirical studies. The text below will then summarize the main findings with regard to each key construct. Publications related to binge eating disorder were included when their findings were deemed likely to influence how EE is understood [52].

**Table 1:** Psychosocial mechanistic/maintenance contributors to emotional eating in obesity

	Definition(s)	Proposed Role(s)	Select Recent Publications
Learning	• Negative reinforcement = behavior increases when it leads to reduction/removal of an aversive stimulus.	• EE historically thought to be maintained due to reductions in negative affect experienced after EE episodes.	[52–57]
	• Eating expectancies = anticipatory process that influences subsequent behavior; developed as a result of previously learned associations.	• Over time, this is theorized to lead to the development of unhelpful eating expectancies (e.g., “eating will reduce negative affect”).	
	• Classical conditioning = unconsciously forming associations between two stimuli, one of which is biologically relevant (e.g., food); the original “neutral” stimulus ultimately leads to a conditioned response.	• If a negative emotion is repeatedly paired with food intake, that emotional state could become a conditioned stimulus, and the desire to eat, a conditioned response.	
Emotion Processing and Regulation	• Emotion regulation = attempts to influence the experience and expression of emotions.	• Difficulties with regulating emotions broadly, or with specific regulation skills, may increase likelihood of using strategies such as emotional eating to cope with distress.	[58–64]
	• Expressive suppression = attempts to hide/reduce emotionally expressive behavior.		
	• Rumination = perseverative, negative thinking about feelings and problems.		
	• Cognitive biases = systematic, distorted thought patterns.		
	• Alexithymia = difficulty identifying, understanding, and expressing emotions.		
Interoception	Interoceptive ability = ability to detect and interpret internal body signals such as hunger or satiety.	• Deficits in interoception may make it more likely for overeating to take place in response to negative emotions.	[65–68]
		• Interoception could play a role in association between emotion regulation and EE, given importance of interoception to emotion processing and regulation	
		• Deficits in interoception could make it difficult to distinguish between hunger and emotional states.	
Stigma/Discrimination	• Stigma = negative attitude toward person or group	• Experiences of stigma/discrimination may increase negative affect and ultimately EE.	[69–76]
	• Discrimination = unfair or unequal treatment based on some aspect of identity or group membership		
		• Experiences of stigma/discrimination may lead to difficulties with emotion regulation and greater EE.	
	• Weight Bias Internalization = negative, self-directed application of weight-related stereotypes		
	• Superwoman schema = schema often developed by women, particularly African American/Black women, to cope with stigma, discrimination, and societal pressure (both helpful and harmful); characterized by strength, resilience, and self-sacrifice		
Dietary Restraint	• Dietary restraint = tendency to restrict/control food intake.	• Efforts to restrain intake over time may actually lead to greater disinhibited eating, such as emotional eating.	[51, 77–80]
	• Cognitive restraint = purposeful effort to control food intake in order to influence weight/shape; at times used interchangeably with dietary restraint.		

*EMA* Ecological Momentary Assessment; *EE* Emotional Eating; *BMI* Body Mass Index

#### Learning

Recent work supports the association between negative reinforcement eating expectancies (i.e., anticipating that eating will relieve negative affect) and disinhibited eating (i.e., eating in response to various stimuli, including emotions) among those with overweight and obesity [53]. At least among college students with obesity, difficulties with emotion regulation and distress tolerance may increase vulnerability to such expectancies [54, 55]. Though the sample focused on those with binge eating disorder (*M*_*BMI*_ = 34.30), recent ecological momentary assessment (EMA) evidence suggests a dynamic process whereby greater reinforcement (affect improves after binge eating) on one day predicts greater affect-regulation eating expectancies the next day (i.e., expecting that eating will improve mood) [52]. Future studies could apply this methodology to examine the role of eating expectancies among those with obesity, without binge eating disorder. Bongers & Jansen [56] highlight the potential role of classical conditioning, having conducted an experimental study wherein participants were conditioned to expect and desire food after experiencing a negative emotion. Having a higher BMI was associated with greater likelihood (marginally significant) of choosing chocolate over money when in a negative mood, compared to no difference in this choice task for those at a lower BMI. The authors proposed that those with obesity might be particularly susceptible to cue-induced responding. Finally, Papalini [57] recently proposed a relief learning model of overeating, suggesting that eating in response to stress may be motivated by the pursuit, and ultimately acquiring, of safety/comfort, in contrast to traditional affect regulation models that suggest EE is maintained by reductions in negative affect. Additional investigations testing whether relief reinforces EE among those with obesity is needed.

#### Emotion Processing and Regulation

A recent review found support for an association between difficulties processing emotions and the desire to eat in response to negative emotions [58]. Though the association between emotion regulation and EE appears to be consistent, regardless of weight status [13], deficits in at least some aspects of emotion regulation appear to be more pronounced among those with obesity. For example, a recent systematic review highlighted increased tendencies toward emotional avoidance and suppression in particular [59]. Others have suggested that compared to those without obesity, those with obesity may have a higher likelihood of experiencing alexithymia [60], as well as reduced attention to and repair of emotions [61], but not greater emotional intensity or lability [61]. This may help to explain the relatively increased risk of EE among those with obesity. Suppression and rumination appear to be particularly important targets for future EE interventions [59, 62, 63]. Finally, a recent laboratory study suggests that future research should explore the role of shame-related cognitive biases in emotional eating among those with or without obesity [64].

#### Interoception

Higher BMI is associated with reduced ability to detect internal states [65], decreased trust in interoceptive information, and less use of this information to guide behaviors [66]. In a recent (albeit small) study, youth with obesity showed reduced interoceptive accuracy on heartbeat task, but enhanced taste perception of sweet foods [67]. Interoception is thought to be intricately linked with emotion regulation [81]; to effectively regulate, one needs to accurately detect changes in emotional and physical cues, and differentiate between the two. Willem and colleagues [68] found that lower interoception was associated with higher levels of EE via reduced interoceptive reliance and higher emotion dysregulation. Interventions targeting EE would be optimized if they not only sought to improve interoceptive ability, but also help patients develop greater trust in that information.

#### Stigma and Discrimination

Accumulating evidence points to an association between weight bias internalization and dysregulated eating behaviors, including EE [69]. Among individuals seeking bariatric surgery, this association appears to be partially explained by shame and (reduced) self-compassion [70]. A recent EMA study highlighted that among those with overweight, experiencing a weight-stigmatizing event was associated with consuming more food [71]. Furthermore, a recent study of 191 mother-daughter dyads suggests the potential importance of interrupting intergenerational processes related to internalized stigma, including body esteem, self-compassion, and EE [72]; follow-up is needed to examine similar processes among caregivers and youth with obesity. Recent work in this area also highlights the need to consider the potential for multiple, intersectional aspects of identity as they relate to experiences of stigma, discrimination, and EE [73–75]. For example, among African American/Black adolescent males, higher everyday discrimination scores were associated with higher BMI via greater endorsement of EE; however, sexual orientation was the largest single predictor of EE [73]. In another recent study, gendered racial microaggressions were associated with greater EE via stronger endorsement of the “superwoman” schema and reduced self-compassion [74].

#### Dietary Restraint

Though included in several theoretical models of EE (e.g., Restraint Theory [82]), few recent investigations of EE and obesity have assessed dietary restraint, and results have been mixed. For example, among U.S. military veterans with overweight/obesity, EE was not associated with dietary restraint behavior nor restraint intention [77]. However, in a sample of women with overweight, the association between frequent dieting and weight gain was mediated by EE [78]. In a study of people pursuing bariatric surgery, higher cognitive restraint pre-surgery was associated with experiencing less change in Hunger post-surgery which predicted less change in EE and ultimately less weight loss at 2 years post-surgery [79]. In a large, international study, participants who scored high on EE also scored higher on dietary restraint, both of which were associated with higher BMI [80]. Notably, a recent meta-analysis of 67 studies reported mixed findings in the literature regarding the association between self-reported dietary restraint and EE, and an overall small pooled effect [51]; this meta-analysis included those across the weight spectrum.

### Aim 3: Behavioral Interventions for EE

Given the psychological and behavioral implications of EE, research has turned toward exploring intervention strategies to ameliorate EE and associated outcomes. A significant drawback to the current emotional eating intervention literature is that most studies do not describe standard intervention protocols which may incorporate concepts from a variety of therapeutic orientations. This makes it difficult to accurately parse out which therapy concepts, approaches, and skills are producing effects on emotional eating. Nevertheless, an overview of the efficacy of interventions by approach (i.e., cognitive behavioral, mindfulness, acceptance and commitment therapy (ACT), and dialectical behavior therapy (DBT)) is described in text below. Additionally, see Table 2 for further description of EE interventions, including mechanism of action and intervention components. For additional recent reviews (which we synthesize below), see Chew and colleagues [93] and Smith and colleagues [87].

**Table 2:** Emotional eating intervention descriptions

Approach	Definition	Mechanisms of Action	Intervention Approaches, Concepts, and Components	Example Therapeutic Exercises
Cognitive Behavioral (CBT)	Psychological and behavioral problems are based on faulty, unhelpful, or incomplete thinking patterns and learned patterns of maladaptive behaviors [83]	● Learning, negative reinforcement ● Maladaptive responses/thoughts, including emotion-related eating expectancies, with adaptive thoughts/behaviors	● Generate adaptive (non-eating) responses to triggering stimuli ● Enhanced coping resources for stress/distress ● Awareness, modification, and prevention of maladaptive thinking and behavior patterns	● Cognitive restructuring ● Stimulus control ● Self-monitoring (e.g., food diary, activity monitoring) ● Goal Setting
Mindfulness	Awareness that arises through “paying attention in a particular way: on purpose, in the present moment, and nonjudgmentally” [84]	● Emotion regulation ● Interoception ● Psychological flexibility ● Self-compassion ● Reward sensitivity	● Interventions: - Mindfulness-Based Stress Reduction (MBSR) [85, 86] - Mindfulness-Based Eating Awareness Training (MB-EAT) [87–89] - Meditation ● Psychological Education (emotions and mindful eating concepts) ● Attention self-regulation (i.e., observation and awareness of thoughts, feelings, and sensations in the here and now) [90] ● Orientation toward one’s experience (i.e., being intentionally curious, open, and accepting of the lived experience) [90]	● Sitting practice in vivo ● Mindful eating (e.g., raisin taste exercise) ● Hunger/fullness ratings ● Loving kindness meditation ● Body scan ● Mindful breathing
Acceptance and Commitment Therapy (ACT)	Life difficulties can arise from psychological rigidity and experiential avoidance (avoiding or attempting to avoid difficult or painful experiences, thoughts or memories) [91]	● Psychological flexibility ● Awareness of internal and external experiences and cues ● Engagement (rather than avoidance) of distressing stimuli or emotional responses ● Behavioral activation toward values	● Mindfulness ● Promotion of acceptance of (i.e., acknowledgement of) the current experience ● Committed action toward (e.g., physical activity) one’s values (e.g., health) ● Diffusion of psychological experiences objectively rather than as threats fused with the self ● Self as context (i.e., psychological experiences and the self as transient and evolving rather than stagnant) ● Values Exploration	● Being present (e.g., 5 senses exercise) ● Observer meditation ● Values identification ● Willingness and action plan
Adapted Dialectical Behavior Therapy (DBT)	Grounded in behaviorism, teaches the principles of radical acceptance and distress tolerance to improve quality of life [92]	Overlap with both ACT and Mindfulness - Learning, negative reinforcement - Emotion regulation	● Mindfulness ● Distress tolerance (i.e., tolerate pain and distress more effectively) ● Emotion regulation (identify and manage emotions more effectively) ● Interpersonal effectiveness (improve communication and relationships)	● Body scan ● Behavior chain analysis ● Mindful breathing ● Opposite action ● DEAR MAN (Describe, Express, Assert, Reinforce, Negotiate) ● Wise mind

#### Cognitive Behavioral Interventions

Cognitive Behavioral Therapy (CBT) is generally considered the “gold standard” approach for the behavioral treatment of obesity [94] and eating disorders (e.g., Binge Eating Disorder) [95]. CBT has been explored as a standalone treatment for EE and as a theoretical approach in behavioral weight loss programs (BWLPs) which also may target change in EE symptoms [87, 93]. CBT is thought to reduce EE by supporting participants in recognition of stressors (and emotions that result) and replacing an urge to use food with more adaptive techniques [87, 96, 97]. For example, self-monitoring of food intake can help increase awareness of antecedents and patterns that impact EE behaviors.

The current literature suggests that CBT is an effective intervention strategy for reducing EE; in fact, Smith and colleagues [87] found that CBT showed the most promise for reducing EE compared to other intervention modalities, although effect sizes were small given the generally short-term nature of many interventions. Across BWLPs (most of which include CBT approaches), results indicate that these interventions produce small to medium reductions in EE immediately after treatment completion, with smaller effects at 3-months post- and no remaining effects at 6-months post-intervention [93].

#### Mindfulness-Based Interventions

The field of mindfulness-based research has grown exponentially since 2006 [98] and mindfulness-based interventions are among the most studied psychological approaches for EE [87] with many interventions utilizing techniques from Mindfulness-Based Stress Reduction or Mindfulness-Based Eating Awareness Training [85–87, 99]. Some of these interventions were designed specifically for people with binge eating [99] but have been adapted and used to target emotional eating [86]. Furthermore, mindfulness components are commonly integrated into a wide variety of interventions including more traditional behavioral approaches like CBT and “third wave” behavioral approaches like ACT and DBT (described below).

Results of mindfulness interventions generally show positive benefits on EE among individuals with or without obesity [86–88, 100–102]. Mindfulness-based interventions have also shown promise in decreasing EE when delivered in primary care [103]. BWLP programs that incorporate mindfulness show larger effect sizes for emotional eating symptom reduction than other combination therapies such as standard BWLP + CBT [93], although results across studies are mixed. Mindfulness-based interventions seem to be more effective in changing EE than weight or eating behaviors in BWLPs [87], although some studies have found that compared to standard BWLPs, adding mindfulness and CBT skills may produce significant, yet mixed results [104, 105]. In the study by Goldbacher et al., weight and emotional eating symptoms significantly decreased in both groups (EE-focused treatment versus standard behavioral weight loss treatment) with no between group differences [104]. In contrast, the study by Corsica, et al. found that across three groups (a modified MBSR intervention, a CBT stress eating intervention, and a combination intervention) perceived stress and stress-eating was significantly reduced with the combination intervention resulting in the greatest treatment effects and moderate weight loss effects [105].

#### Acceptance and Commitment Therapy (ACT)

ACT is considered a third-wave CBT that has shown efficacy in the treatment of obesity [106] and has been studied as an intervention for eating disorders and body image concerns [107, 108]. Several studies have suggested the benefit of incorporating ACT interventions to reduce EE; ACT interventions may be slightly less effective than CBT, yet this literature base is emerging and remains somewhat mixed [87]. Even a 1-day ACT intervention may provide benefit for those experiencing EE [108]. However, another recent study explored the efficacy of an ACT intervention for disinhibited eating and found no additive benefit of ACT compared to standard care [109].

#### Adapted Dialectical Behavioral Therapy

DBT is another third-wave approach that has been examined as an EE intervention in adapted formats. A group-based, adapted DBT skills intervention has been validated to treat binge eating disorder [110]. Given that binge eating disorder and EE may exist along an “eating continuum” with overlapping deficits in emotion regulation and inhibition [13], this intervention modality likely holds promise for EE symptom reduction as well. Two, non-randomized pilot studies examined DBT skills interventions as a treatment for EE among people with obesity and both studies showed preliminary evidence of acceptability and efficacy [111, 112].

## Conclusion

The present review revealed several key findings specific to EE among people with obesity. First, contemporary research, which includes a small body of longitudinal studies, substantiates early research (e.g., Ganley [14]), showing an association between obesity and EE. Furthermore, individuals with obesity and EE are also likely to report more unhealthy dietary habits, higher levels of depression, anxiety, and stress, and more disordered eating. In reviewing research on psychosocial mechanisms and maintenance factors, the most robust literature exists in support of principles of learning and emotion regulation. Also of note, dietary restraint is rarely incorporated in recent empirical studies, despite early dominance in research on EE and obesity (e.g., restraint theory [21]).

The literature on behavioral interventions for EE is emerging and can be seen through two lenses: interventions targeting EE directly and interventions targeting weight loss (i.e., BWLPs) that may also impact EE symptoms. Although weight management interventions have traditionally mostly excluded a focus on EE [113], the present review emphasizes that contemporary weight loss approaches are often based on theory that recognizes the role of emotion regulation, interoception, and psychological flexibility, in addition to traditional behavioral mechanisms. Nonetheless, most of these interventions are in the context of weight management and do not necessarily directly target EE, even when it is measured as an outcome. Despite this, behavioral interventions using a variety of approaches can produce positive reductions in EE, although efficacy and durability vary by study and are not always reported. CBT was the most effective approach for EE [87]. The literature on BWLPs and EE is mixed, yet the most recent review and meta-analysis [93] found small to moderate effects for EE symptom reduction at post-treatment and 3-months post-treatment, with the Mindfulness-Based Eating Awareness Training program having the greatest effect size (*g* = 0.86).

### New Insights

#### Association Between Obesity and EE

Systematic research on EE has been conducted for more than 3 decades [14]. In addition to bolstering key findings from early research, the present review revealed novel, recently published research findings, or new insights, related to the study of EE in obesity. Some of this work adds important nuance to foundational research on the relationship between and associated risks of EE and obesity. For example, recent literature shows that EE is elevated in individuals with obesity, but rates of EE between healthy weight and overweight are not significantly different [32]. Researchers and clinicians should be mindful that while emotional eating occurs across all body sizes and can to some degree reflect normative coping, elevated levels may be more pronounced in individuals with obesity compared to those with overweight. With that, when EE occurs in people who do not have obesity, it may not be indicative of considerably poor dietary patterns (e.g., greater snacking, intake of energy-dense foods); but, in the context of obesity, EE is related to unhealthy dietary intake [47].

Other, recent research has shed light on unique emotions as they trigger eating. In a clinical sample, boredom was the most common emotion to trigger eating, but sex differences were observed [114]. Compared to men, women endorsed higher levels of eating in response to depression, anger (as measured via subscales) and feeling blue and upset (as measured via individual items). Unique patterns between distinct emotions and eating may also vary based on obesity treatment since these sex differences were only observed in participants who were treated with obesity medication or surgery as opposed to lifestyle modification, only. While it is clear that people with obesity are at risk for emotional eating, continued work that focuses on clarifying additional factors that indicate for whom and when the obesity, EE link occurs, will help inform prevention and intervention research.

#### Theoretical Underpinnings

The newest theoretical research builds upon learning theory, provides more clarity on the role of emotion regulation, and highlights interoception and stigma/discrimination as salient theoretical variables. In a recent opinion paper (2024), the author integrates previous overeating theories, address shortcomings, and use learning theory to explain how emotional eating may develop by providing a sense of comfort and relief [57]. This model, which has yet to be tested, offers a contemporary, unique explanation for EE that should be examined in future empirical investigations. Qualitative research reveals a more detailed understanding of escape theory that may inform further research and intervention development [63]. In this small sample, EE occurred when individuals were in a state of “narrative focus” in which they were thinking about the past or future, sometimes coinciding with ruminative thinking. When engaging in EE, participants reported enjoying the positive sensory experience and a present moment focus. Other research closely examines interoception as a contributing factor to EE [67], emphasizing specific aspects of interception such as noticing bodily sensations, reliance on bodily sensations [68], and awareness of appetite and emotions [115]. New research conducted with African American/Black women emphasizes the significance of considering sociocultural factors in our understanding of the development of emotional eating ([74, 75, 116]). Stress due to experiencing gendered, racial microaggressions and the strong black woman schema (i.e., cultural expectations of Black women to be strong, independent, and resilient) are significantly, positively associated with EE. This work should be further investigated in the context of obesity, in addition to expanding EE research to other minoritized populations.

#### Intervention Research

Cutting edge research on interventions for EE includes medication and behavioral trials. Given the sharp increase in the use of GLP-1 medications for weight loss, continued, empirical study is needed to examine whether these medications simultaneous impact problematic eating behaviors, such as EE. In one sample of adults being treated with semaglutide in conjunction with a lifestyle intervention, the proportion of emotional eaters significantly decreased from 72.5 to 11.5% over a 3-month period [117]. With regard to behavioral research, since publication of the Chew and Smith systematic reviews, only two behavioral intervention studies focused on EE have been published [103, 112]. Morillo-Sarto and colleagues conducted an RCT in which adults with overweight/obesity were randomly assigned to usual care or a 7-week mindfulness-based intervention specifically designed to treat EE [103]. Compared to usual care, the mindfulness intervention was associated with significant improvements in EE at post-treatment (small effect size) and follow-up (medium effect). The authors note that one possible reason for the encouraging results is that unlike previous mindfulness-based approaches that emphasize general mindfulness practice, their program specifically focused on mindful eating. Despite positive change in EE, no change in weight/BMI was observed. In another recent open pilot study, Braden and colleagues [112] delivered a 16-week intervention for adults with EE and obesity. In their study, the first 10 sessions of the intervention were solely focused on targeting EE via DBT skills, and then traditional BWL techniques were introduced in the final 6 sessions. Large effects were observed for EE and BMI change. The study has several limitations, including a non-randomized design, but preliminary results appear promising. Both of these novel interventions highlight the possible efficacy of behavioral treatments that specifically target EE, distinct from weight loss.

Limitations of the existing literature highlight important areas for future research. First, although this review focused on eating in response to negative emotions, it is important to recognize the need for future research to assess under-eating in response to negative emotions [118], in addition to eating in response to positive emotions [119]. Another important limitation relates to jingle/jangle fallacies [120], wherein constructs that are highly similar are labeled as separate, or alternatively, dissociable constructs are given the same label. This was most apparent in the literature on emotion processing and regulation and intervention mechanisms and concepts, but is also notable across studies of EE, as the outcome itself is variably defined [121]. Likewise, a great deal of intervention overlap exists (e.g., between CBT and Acceptance-based interventions, mindfulness is a core component of ACT and DBT). There is a need for dismantling studies to aid our understanding of the core effective components of these interventions. Two related challenges are the history of mixed findings and the issue of directionality. Most of the cited work utilized cross-sectional designs. Thus, it remains unclear whether deficits in emotion processing precede or are a consequence of obesity [60]. Mixed findings were most apparent in the literature examining the role of dietary restraint in EE. As opposed to being a clear cause-effect relationship, this association is likely bi-directional and moderated by psychological (e.g., emotion regulation, cravings) and contextual factors (e.g., presence of others). Another key issue— since EE studies often to not assess the amount of food consumed, we are unable to tease apart the impact of EE, distinct from overeating and binge eating. Likewise, EE eating is sometimes studied in the context of binge eating, and it is not always clear how/whether this literature applies to EE among those without BED. Regarding intervention studies specifically, most studies were conducted in the United States, samples were predominantly female, and a large proportion of studies did not report on participant ethnicity, which represents a major gap in the literature. Finally, few studies explored EE outcomes at and beyond 12-month post-intervention. Greater focus on long-term intervention effects on EE is needed.

Obesity is a heterogeneous condition with a multifactorial etiology, which includes subtypes other than EE (e.g., abnormal satiation [2]). Nevertheless, the current review describes EE as a common experience among adults with obesity. In considering psychosocial contributors to EE, evidence supports emotion regulation deficits and behavioral principles of classical and operant conditioning and eating expectancies as central theoretical factors. Treatment findings are somewhat encouraging given that behavioral interventions are associated with improvements in EE in the short-term but could be optimized to better align with the literature on mechanisms of EE and focus on components that are most efficacious, either alone or in combination. Ongoing study of EE in a variety of clinical populations is needed. Additionally, development of innovative treatment approaches that effectively target established EE mechanisms, such as emotion regulation, will be important for improving outcomes for those with obesity who are experiencing negative consequences of EE.

## Key References

•• Chew HSJ, Lau ST, Lau Y. Weight-loss interventions for improving emotional eating among adults with high body mass index: A systematic review with meta-analysis and meta-regression. Eur Eat Disord Rev. 2022 Jul;30(4):304–327. doi: 10.1002/erv.2906. Epub 2022 Apr 23. PMID: 35460323; PMCID: PMC9320927.

This paper is a recent review and meta-analysis summarizing the effects of behavioral weight loss programs (BWLPs) on emotional eating symptoms among adults with obesity. Results suggest small to moderate effects of BWLPs on emotional eating.

•• Chew HSJ, Soong RY, Ang WHD et al. (2025) The global prevalence of emotional eating in overweight and obese populations: A systematic review and meta-analysis. Br J Psychol.

This systematic review and meta-analysis examined the prevalence rates of emotional eating in the context of overweight/obesity or weight gain. Results showed a prevalence rate of 44.9% when emotional eating is measured using self-report questionnaires.

•• Dakanalis A, Mentzelou M, Papadopoulou SK et al. (2023) The association of emotional eating with overweight/obesity, depression, anxiety/stress, and dietary patterns: a review of the current clinical evidence. Nutrients 15(5):1173.

This systematic review showed links between emotional eating and overweight/obesity, depression, anxiety, stress, and dietary patterns with a focus on studies from the past 10 years.

•• Smith J, Ang XQ, Giles EL, Traviss-Turner G. Emotional Eating Interventions for Adults Living with Overweight or Obesity: A Systematic Review and Meta-Analysis. *Int J Environ Res Public Health*. 2023;20(3):2722. Published 2023 Feb 3. doi:10.3390/ijerph20032722.

This systematic review and meta-analysis summarizes the recent literature on behavioral interventions for emotional eating. Results showed benefit of a variety of intervention modalities on emotional eating symptoms with Cognitive Behavioral Therapy (CBT) showing the most promise.

•• Vasileiou V, Abbott S (2023) Emotional eating among adults with healthy weight, overweight and obesity: a systematic review and meta-analysis. Journal of Human Nutrition and Dietetics 36(5):1922–1930.

This meta-analysis showed that there are no differences in reported emotional eating between adults with a healthy weight and overweight status. Adults with obesity reported significantly more emotional eating that adults with a healthy weight status.

• Fuente González CE, Chávez-Servín JL, de la Torre-Carbot K et al. (2022) Relationship between emotional eating, consumption of hyperpalatable energy-dense foods, and indicators of nutritional status: A systematic review. Journal of obesity 2022(1):4243868.

This systematic review showed that emotional eating is associated with greater consumption of high fat/high calorie foods across the lifespan.

• Hunger JM, Montoya AK, Edrosolan K et al. (2024) Ecological momentary assessment of weight stigma and eating behavior in everyday life. Annals of Behavioral Medicine 58(6):457–462.

EMA study highlights that within participants with BMI > 25, experiencing weight-stigmatizing events was not associated with being more likely to eat, but was associated with eating a larger quantity of food. The authors suggest that this could trigger a cycle where weight stigma leads to increased eating and future weight gain, and potentially increases risk for future weight-related stigma.

• Papalini S (2024) Stress-induced overeating behaviors explained from a (transitory) relief-learning perspective. Physiol Behav 287:114707.

Proposed a relief learning model of emotional eating, integrating principles of learning with neurobiological evidence related to reward. This theory proposes that eating in response to stress may be motivated by the pursuit, and ultimately acquiring, of safety/comfort, in contrast to traditional affect regulation models that suggest emotional eating is maintained due to a reduction in negative affect.

• Volpe VV, Collins AN, Ross JM et al. (2024) Black young adult superwomen in the face of gendered racial microaggressions: Contextualizing challenges with acceptance and avoidance and emotional eating. Annals of Behavioral Medicine 58(5):305–313.

In a study of 504 Black, young adult women, they highlight the importance of an intersectional lens and culturally sensitive interventions in emotional eating-related work. They found that endorsing a superwoman scheme, avoidance, and acceptance all played a role in the association between gendered racial microaggressions and emotional eating.

## Data Availability

No datasets were generated or analysed during the current study.
